# A Lecture on Caries and Necrosis of Bone

**Published:** 1868-02

**Authors:** H. R. Noel

**Affiliations:** Professor of Physiology.


### ARTICLE II.

### *A Lecture on Caries and Necrosis of Bone,*

Delivered Nov. 15, 1867, at the Baltimore College of Dental Surgery,

(Concluded.)

By H. R. Noel, M.D., Professor of Physiology.

Now as regards the phenomena of necrosis, Dr. Beale is equally unfortunate in his explanations. Dr. Beale says in necrosed bone, (*from occlusion of blood vessels* as he gives no other cause), the nutrient fluid not being able to pass through its regular channels, passes *around* and passes rapidly, so the germinal matter around, is here also too freely supplied with nutrient material. He thus explains the rapid production of germinal matter, cells, half formed tissue, &c., which rises around and invests the bone. A carious condition induced. This is another specimen of gratuitous assertion not sustained by the facts of the case.

An Haversian canal becomes occluded, and the fluid which should pass through, passes around it,—and all of the phenomena, of caries, &c., are produced by this excess of nutrient fluid in the tissue surrounding.

This involves two assumptions, the one a new idea and the other the view we have examined and refuted.

(1.) It implies that each tissue has its amount that passes through regularly in a given time, and if therefore it cannot get through by the usual channels, *it* must get through by others, and to do so, it makes up by rapidity, what it has lost in directness of rout.

This is rich, and when applied to secretions of saliva bile—milk, &c., gives us a rare physiological explanation of variable action in glands. It implies that the heart propelling the blood, sends amounts through tissues, determined by the size of the arteries, and ignores certain other forces concurred in the circulation. He gives the vis-a-tergo—but says nothing of the "vis-a-fronte."

Now in this very case we assert that the circulation is increased and *determined* by the "vis-a-fronte," and not by the "vis-a-tergo."

(2.) Assumption of premises and the deduction of conclusions therefrom is, as we have said—any thing than convincing.

But does excess of nutrient fluid determine the excessive production of germinal matter, &c., in necrosis? We think not.

In the ligature of the main artery of a limb, say the femoral, we have on a greatly magnified scale, the same condition of things that obtains when an Haversian canal is occluded. The current is obstructed in the main channels, seeks divergent and new channels, passes around through the tissues, converges beyond the ligatures and reenters the old channel below the ligature.

Here the germinal matter of the surrounding tissue has 50 or 100 times as much nutrient fluid passing it as is normally passed, and we would expect, if Dr. Beale's theory obtained, to have all the phenomena of caries on a grandly exaggerated scale.

The collateral circulation is indeed greatly increased; but you will look in vain for the phenomena of *caries.* Œdema you may have, but the rapid increase of germinal matter the parasitical attack upon formed tissue, you do not find, certainly it is *never present* as a direct effect of such a cause.

Dr. Beale is painfully unfortunate in this assertion as in the others. Dr. Beale in assigning the causes of necrosis, gives in reality only one.—Viz.: "cutting off of the nutrient fluid." No one will deny, but that this cause is adequate to the production of death of the germinal matter in bone, or any other tissue ; but are we to understand then, that *this* is the only cause capable of producing necrosis? may not an agent poison the germinal matter ? Cannot the vitality of a part be destroyed, while abundance of fluid is circulating around it? We answer this affirmatively of course. Hence necrosis may be caused by an agent acting directly upon the vitality of the germinal matter. Concentrated sulphuric acid would do it by chemically disorganizing it, and we believe that there are agents which act directly upon the vitality of the germinal matter. Gangrene and mortification may occur, with all vessels patulous and nutrient fluid flowing through them. And if this be true of the soft tissues, it is true also of bone.

So there may be necrosis of bone with *open canals,* and the blood therefore *not be necessarily compelled* to go around the dead bone. The fact is, it does go around, but not because there is any physical obstruction of the canal, not because it *cannot* but because it *will* not, go through dead bone. It *can* but *will not.* It is not needed there and therefore it does not go through—the "vis-a-fronte" is destroyed.

The heart though the great propelling power, is by no means the only force concerned in the circulation ; there are several others, and one among the most powerful and constant is the affinities developed in the chemico-vital changes of nutrition, assimilation, &c. To be very plain in illustrating, we would say that the secretions of saliva, milk, bile, &c., show us that when blood is needed in an organ or tissue it is *sent* there; or it is *drawn* there, and the amount flowing thither is measured by the demand made.

Now we wish to distinctly affirm, that the germinal matter, cells, loose tissue, &c., which is produced around, is not caused by the too rapid flow of blood or nutrient fluid, which, once passed through, but now has to pass around the necrosed bone; but that the rapid transformations occurring in the production of germinal matter—cells, loose tissue, &c., require an extra amount of blood and that extra amount is *sent* there, or is *drawn* there.

The first steps are the vital changes, the next is the demand for more blood, and the third and last is the increased flow of blood to the part. Dr. Beale has exactly inverted the true order, and place the effect as the cause.

We have taken up every point of Dr. Beale's theory—we have carefully examined each one, and we unhesitatingly pronounce it as wild and as visionary as he could well have made it.

Nothing in nature—nothing in physiology—nothing in pathology sustain it. Utterly fallacious.

The true theory of Caries and Necrosis.

We believe that the true theory of caries, is one of "lowered vitality"—that the true theory of necrosis is *death* of the germinal matter in the tissue and death of the tissue also. You perceive that the cause may be the same, the degree or intensity of its action determining the result as to either caries or necrosis.

But how can the theory of lowered vitality, be reconciled with the rapid increase of germinal matter, cells and loose tissue? We will endeavor to show, that it is the exact condition that will account for it,—and that upon no other grounds can a satisfactory explanation be given.

And here we shall follow that admirable practical physiologist, Dr. T. K. Chambers, to whose lectures upon mucus and pus we have before referred.

You remember that Dr. Chambers begins the formation of mucus, by the arrest of development of a "mucous globule" (i. e. a small mass of germinal matter) upon or near the basement membrane of a mucous membrane, and pus is but a continuation of this same process. This "globule" has its vitality lowered, does not develop into an epithelial cell as it should do, but still retains enough of vital force to transform pabulum and to multiply by division, budding, &c. A few globules rapidly multiplying would use up a good supply of pabulum, and yet might form no well developed tissue ; in fact might produce only germinal matter, cells and loose tissue in excess ; or even mucus or pus. This is the true explanation of profuse suppuration. The vital power when too low, to exhibit the vital phenomena of transformation into tissue, may yet have the power of reproduction and power of transformation of pabulum into germinal matter, and we would have no well matured tissue, but an excessive amount of germinal matter, mucus or pus.

These changes progressing, and the pabulum being consumed, more is demanded—and more is obtained—hence the phenomena of increased circulation. This increase in circulation being the *result* of the active change going on, but, by no means the *cause* of these changes.

We quote the following from Chamber's lectures "Renewal of life," pps. 72 and 73.

"Is rapidity of multiplication to be looked upon as evidence for or against the force of vitality ? Against I think.

The lower we go in the scale of creation, the more quickly and the more copiously, do the living forms representing the various classes reproduce their kind.

The less function and force and intensity of existence they have, the more prominent becomes reproduction as the main object of their being created. This seems to be the universal rule to be traced all through living beings, till we get down to the Amœba and the mould, in which *no trace of a function* can be detected beyond the multiplication of their substance.

And this is the law that obtains in the production of mucus—pus and even tissue; this is the true solution of the phenomena of caries. The cause of caries is not an excess of pabulum but deficient vitality. The germinal matter reproduces itself and multiplies rapidly in quantity and does not form tissue, not because of an excess of nutrient fluid, but because its vital power is lowered. It is devitalized and lacks intensity, and shows only the lowest form of vital force i. e. that of reproduction.

The decomposition of necrosed bone may give rise to chemical compounds, which exert a depressing or devitalizing influence upon the tissues and their germinal matter, this might and would cause all the phenomena described by Dr. Beale. We would have a rapid reproduction of germinal matter, cells and loose tissue, as the direct result of lowered vitality; and also an increased circulation around the necrosed bone produced by the increased demand for pabulum to support these active changes.

Caries and necrosis are therefore only different degrees of the same—devitalization. Caries is lowered vitality—necrosis is death. The cause acting might produce death in one part, and lowered vitality around, hence the two are so often found combined, being but different degrees of the same thing and dependent therefore upon the same cause.

The intensity of action of the cause, and the strength or feebleness of the part determining which condition shall obtain.

We conclude therefore that caries and necrosis are caused by agents acting upon the vital powers of the bone tissue, and that the attendant phenomena are fully explained by the views of Dr. Chambers.

That the "theory of Dr. Beale" is unphilosophical, unphysiological and superficial, and based upon assumptions which are false in fact and do him no credit as a physiologist.

